# OmpR and RcsB abolish temporal and spatial changes in expression of *flhD* in *Escherichia coli* Biofilm

**DOI:** 10.1186/1471-2180-13-182

**Published:** 2013-08-02

**Authors:** Priyankar Samanta, Emily R Clark, Katie Knutson, Shelley M Horne, Birgit M Prüß

**Affiliations:** 1 Department of Veterinary and Microbiological Sciences, North Dakota State University, Fargo, ND 58108, USA

**Keywords:** *Escherichia coli*, Biofilm, Reporter gene fusion, Fluorescence microscopy

## Abstract

**Background:**

Biofilms are communities of bacteria that are characterized by specific phenotypes, including an increased resistance towards anti-microbials and the host immune system. This calls for the development of novel biofilm prevention and treatment options to combat infectious disease. In *Escherichia coli*, numerous global regulators have been implicated in the control of biofilm associated cell surface organelles. These include the flagellar regulator FlhD/FlhC, the osmoregulator EnvZ/OmpR, and the colanic acid activator RcsCDB. Using flow cell technology and fluorescence microscopy, we determined the temporal expression from *flhD*::*gfp*, *ompR*::*gfp*, and *rcsB*::*gfp* in *E*. *coli* biofilm, as well as the impact of the negative regulation of *flhD* by OmpR and RcsB. Spatial gene expression was investigated from *flhD*::*gfp*.

**Results:**

The temporal gene expression profile for *flhD* yielded an early peak at 12 h, a minimum of expression at 35 h, and a second increase in expression towards 51 h of biofilm development. In contrast, the *ompR* profile showed a peak at 35 h. A mutation in *ompR* abolished time dependence of *flhD* expression after the initial growth period of 12 h. Intriguingly, *rcsB* expression did not correlate inversely with *flhD* expression, yet a mutation in *rcsB* abolished time dependence of *flhD* expression as well. Spatially, expression of *flhD* was highest in the outermost layer of the biofilm in the parent strain. In *ompR* and *rcsB* mutants, *flhD* was expressed throughout the biofilm. Mutations in both, *ompR* and *rcsB* increased *flhD* expression throughout all temporal and spatial experiments. This increase was paralleled by reductions in biofilm amounts at four tested time points.

**Conclusion:**

Our data lead to the conclusion that FlhD/FlhC and its regulation by OmpR and RcsB may be our first target mechanism for the development of novel biofilm prevention and treatment techniques.

## Background

Bacterial biofilms are defined as sessile communities of bacteria that form on air-liquid or liquid–solid interfaces, or even intracellularly [1]. Due to their high resistance to any attempts of removing them, biofilms have a profound impact in many clinical settings, including catheter-associated urinary tract infections [2], periodontitis [3], and otitis [4], as well as *Pseudomonas aeruginosa* infections of cystic fibrosis patients [5]. Much research has been done on disease mechanisms relating to the biofilm lifestyle. Yet, many of the early studies do not consider that growth conditions for the bacteria differ across the biofilm and also change with time. As one example, bacteria residing within the fully matured biofilm have limited access to nutrients and oxygen, but are also well protected from anti-microbials, as well as the host immune system. In contrast, bacteria that grow at the surface of the three-dimensional structure or are still in the early phases of biofilm formation would have better access to nutrients and oxygen, but are also more exposed to anti-microbials. Some temporal studies of gene expression in biofilms were done years ago [6]. Spatial studies have been done more recently. These were facilitated by advances in microscopy techniques, as well as the development of fluorescent probes [7-9].

Fusions of gene promoters to the structural genes of fluorescence proteins were used to study heterogeneity in biofilms of several bacterial species. This was done to measure: i) spatial gene regulation in biofilm of *Bacillus subtilis*[10], ii) real-time spatial gene expression in *Geobacter sulfurreducens* electricity producing biofilm [11], iii) quantitative gene expression in biofilm of *Salmonella*[12], iv) single cell gene expression in *B*. *subtilis* biofilm [13], and v) the effect of inhibitors on *Pseudomonas aeruginosa* biofilm [14]. To reduce complexity and facilitate genetics experiments, flow cell technology was developed to grow the biofilm [8,15]. This allows the biofilm to form under continuous hydrodynamic conditions at a controlled and reproducible flow rate. In this study, we used promoter fusions to green fluorescence protein (GFP), flow cell biofilms, and fluorescence microscopy to measure temporal and spatial expression of selected biofilm associated genes in *Escherichia coli* biofilms.

The genetic system that is used for this study consists of the flagellar [16] and global regulator [17-19] complex FlhD_4_/FlhC_2_[20] and the two-component systems for osmoregulation EnvZ/OmpR [21] and colanic acid activation RcsCDB [22]. These three regulatory systems are part of a partial transcriptional network that centered around FlhD/FlhC and regulated all the biofilm associated cell surface organelles [23]. In particular, OmpR and RcsB in their phosphorylated form are inhibitors of *flhD* expression [24]. RcsB and OmpR are regulators of type I fimbriae [25,26], as well as expression of many other genes [27,28]. In planktonic *E*. *coli*, growth phase dependent expression of *flhD* required OmpR. Additionally, *flhD* expression in the *ompR* mutant was much higher [29]. This was also true for *flhD* expression and swarming of *Xenorhabdus nematophila*[30].

While all the above research involving OmpR, RcsB, and FlhD/FlhC was done with planktonic bacteria, this study investigates the impact of this regulation on biofilm formation. In particular, we wanted to accomplished three goals: i) provide proof of concept that the study of temporal and spatial expression of biofilm associated genes can lead to the identification of novel targets or target mechanisms for the development of biofilm prevention techniques (gene is expressed early in biofilm development) and treatment options (gene is expressed late and at the edge of the biofilm); ii) attempt to identify FlhD/FlhC as the first such targets, because it is a transmitter between numerous environmental conditions and many cellular responses, and iii) establish OmpR and RcsB as control mechanisms that can be taken advantage of to increase *flhD* expression and reduce biofilm amounts.

## Results

### Temporal gene expression of *flhD*, *ompR*, and *rcsB in E*. *coli biofilm*

#### Expression of *flhD* peaked at 12 h and increased again towards 51 h of biofilm formation

**Figure 1:**
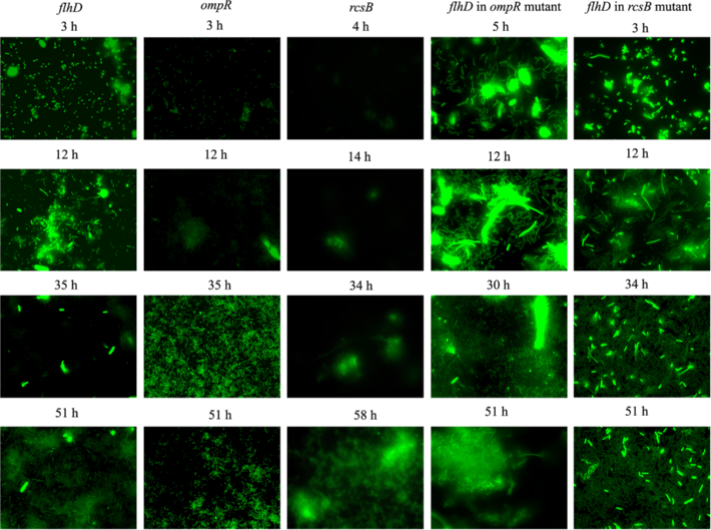
**Fluorescence images of *flhD::gfp, ompR::gfp, rcsB::gfp *in AJW678 and *flhD *in the *ompR *and *rcsB *mutant strains.** Biofilms of BP1470, BP1432, BP1462, BP1531, and BP1532 were grown in flow cells and subjected to fluorescence microscopy. Four time points were selected for each strain; these are printed on top of the respective images. At the very top of each column, promoter names are printed. Images were taken at 1,000 fold magnification.

Fluorescence microscopy images were produced from flow cell grown biofilm of the *E*. *coli* genetic parent strain AJW678 that contained the *flhD*::*gfp* fusion plasmid, called pPS71. Fluorescence signals obtained from these biofilms were highest at 12 h, lowest at 35 h, and then increased again towards 51 h of biofilm formation. This was seen in all four time series of images that had been taken from four independently formed biofilms. A selection of images from one of these experiments is shown in the left column of Figure 1. Occasionally, we observed high signals in individual bacteria of the 3 h sample, but the number of bacteria on the slides was not indicative of a biofilm at that point in time.

**Figure 2:**
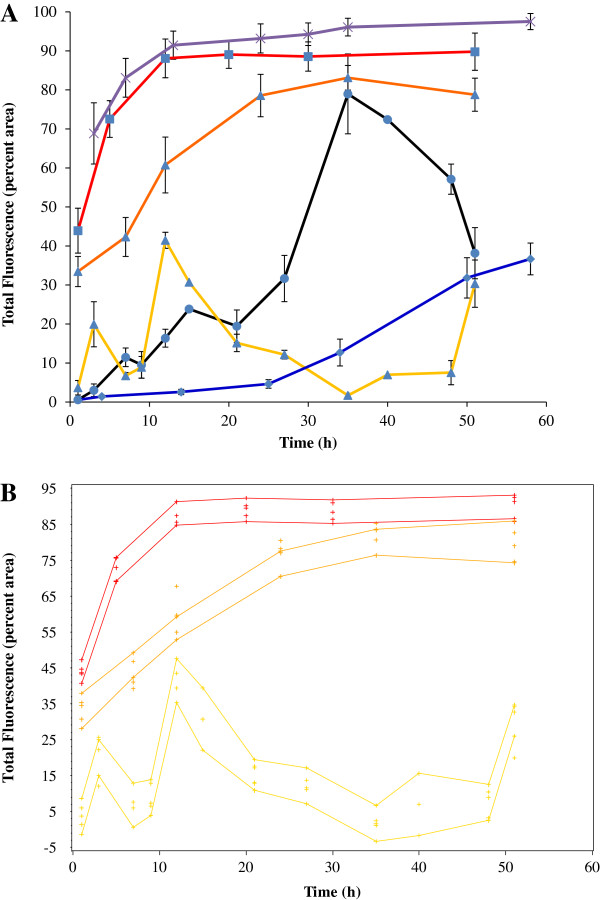
**Temporal expression of *flhD, ompR, rcsB *in AJW678 and *flhD *in the *ompR *and *rcsB *mutant strains. A.** Fluorescence was quantified as percent area of the images that were fluorescent, averages and standard deviations were determined. The x-axis indicates the time (hours) of biofilm formation. The y-axis indicates the total fluorescence intensity in percent area for the different strains at the different time points. The yellow, black, and blue lines are showing the gene expression profile of BP1470 (AJW678 *flhD*::*gfp*), BP1432 (AJW678 *ompR*::*gfp*), and BP1462 (AJW678 *rcsB*::*gfp*), respectively. The red line is the temporal expression profile of BP1531 (*flhD*::*gfp ompR*::*Tn*10), the orange line that of BP1532 (*flhD*::*gfp rcsB*::*Tn5*). The purple line is our housekeeping strain BP1437 which contains the *aceK*::*gfp* fusion plasmid. **B.** Confidence bands were calculated using the loess procedure. Upper and lower lines of each colors are indicating the highest and the lowest level of the total fluorescence intensity. The color code is identical to A.

The images from Figure 1 were converted into quantitative data by calculating the percent area of the images that were fluorescent. The resulting expression profile for *flhD* showed a peak at 12 h (Figure 2A, yellow line, blue triangles). Fluorescence was lowest at 35 h and increased again towards 51 h. We also noticed a small single point peak at 3 h, which is in agreement with the occasional high fluorescence of small numbers of individual bacteria that was visualized on the images (Figure 1). Since fluorescence from the green fluorescence protein reporter is indicative of *flhD* expression, we conclude that *flhD* expression was highest at 12 h, lowest at 35 h, and increased again towards 51 h.

#### The temporal expression of *ompR*, but not *rcsB,* correlated inversely with that of *flhD*

Expression of the negative regulator of *flhD* expression, OmpR, exhibited a temporal profile (Figure 1, second column from the left and Figure 2A, black line, blue circles) that was almost the inverse of *flhD* expression between 21 h and 51 h of biofilm formation. Specifically, *ompR* expression increased between 21 h and 34 h, while *flhD* expression decreased. Between 34 and 51 h, *ompR* expression decreased, while *flhD* expression increased. Expression of another negative regulator of *flhD* expression, RcsB, did not correlate with the temporal expression profile for *flhD* (Figure 1, center column and Figure 2A, blue line, blue diamond’s). Until 25 h, the fluorescence signal from the *rcsB*::*gfp* plasmid containing strain was very weak, but increased steadily after this point in time.

#### Mutations in *ompR* and *rcsB* abolished temporal differences in *flhD* expression

The fluorescence signals from *flhD*::*gfp* in the *ompR* and *rcsB* mutant strains were higher than those from the other strains at all times. Expression of *flhD* in the *ompR* mutant increased over the first 12 h and reached a steady state level after that (Figure 2A, red line, blue squares). Between 12 h and 24 h, expression of *flhD* in the *rcsB* mutant (Figure 2A, orange line, blue triangles) increased more slowly than in the *ompR* mutant, but was reasonably growth phase independent after 24 h as well. This slower increase in *flhD* expression in the *rcsB* mutant (relative to the *ompR* mutant) correlates with the reduced increase in *rcsB* expression (blue line) during the same time period, relative to the increase in *ompR* expression (black line). Statistical analysis of the data with the Loess procedure yielded confidence bands for the *ompR* and *rcsB* mutant strains that did not overlap with that of the parent (Figure 2B).This indicates that there is indeed a statistically significant difference between the parent strain and either of the two mutants.

In comparison, the expression profile for our housekeeping strain that contains the *aceK*::*gfp* fusion plasmid was high at all times (Figure 2A, purple line, cross symbols). Expression increases in any strain during the first 12 h can be explained by the increase in bacterial cell numbers during the early development of the biofilm.

### Spatial gene expression of *flhD* in *E. coli* biofilm

**Figure 3:**
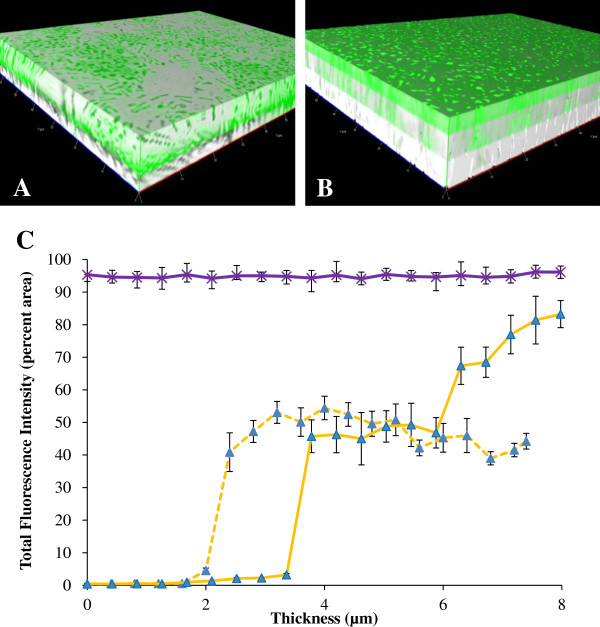
**Spatial gene expression of *flhD *in the parent strain. (A)** and **(B)** are the 3D images constructed from the *z-*stacked images (bright field and fluorescence) at 12 hours **(A)** and 51 hours **(B)**, using BP1470 (AJW678 pPS71). **(C)** is the quantitative representation of the spatial gene expression of *flhD* at 12 hours (dashed yellow line) and 51 hours (solid yellow line) of biofilm formation. The purple line is the spatial expression profile from the *aceK*::*gfp* fusion at 34 h.

From the temporal gene expression experiment, we knew that the highest expression of *flhD* was at 12 h and 51 h of biofilm formation. As a consequence, we performed the spatial gene expression experiment for *flhD* at those two time points. In both the 12 h (Figure 3A) and 51 h (Figure 3B) biofilms, the expression of *flhD* was highest at the outer layer of the biofilm. Fluorescence calculated from the individual images of the *z*-stacks showed that at 12 h, there was little or no expression of *flhD* within the first 2 μm from the surface that the biofilm had formed on (dotted yellow lines). Expression increased rapidly at 2 μm to approximately 50% coverage. In 51 h biofilms, there were three distinct intensity levels (solid yellow lines). Until 3 μm, the expression of *flhD* was very low; at 3.5 μm, the expression jumped to 50% and maintained this level until 6 μm; across the upper 2 μm of our biofilm, *flhD* expression increased to approximately 75% of the total area of the images. Our housekeeping gene in comparison was highly expressed all throughout the biofilm (purple lines).

**Figure 4:**
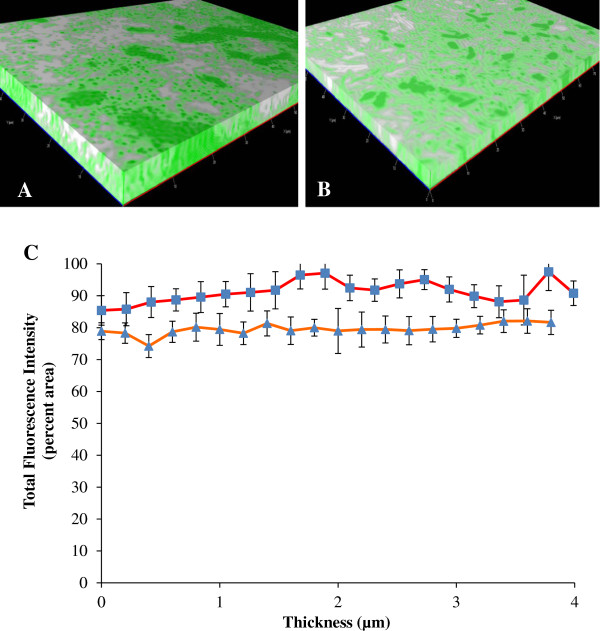
**Spatial gene expression of *flhD *in the *ompR *and *rcsB *mutant strains. (A)** is the 3D image of the 33 h biofilm from BP1531 (*ompR*::*Tn*10 pPS71), **(B)** is the respective image from the 51 h biofilm from BP1532 (*rcsB*::*Tn*5 pKK12). **(C)** is the quantitative representation of the spatial gene expression of *flhD* in the *ompR* mutant (red line) and the *rcsB* mutant (orange line) at the times points represented in **A** and **B**.

The temporal gene expression study had determined that the expression of *flhD* in the *ompR* and *rcsB* mutant strains was constitutively high throughout the experiment after a primary increase during the initial time period of biofilm formation. As time points for the spatial experiment, we selected 33 h for the *ompR* mutant (Figure 4A) and 51 h for the *rcsB* mutant (Figure 4B). Interestingly, expression of *flhD* in both mutants was high across all layers of the biofilm. Fluorescence was between 80 and 95% coverage across the entire biofilm of both mutants (Figure 4C). By all appearances, both OmpR and RcsB abolished spatial differences in *flhD* expression together with temporal ones, while increasing overall expression.

### Mutations in *ompR* and *rcsB* reduced biofilm biomass

**Figure 5:**
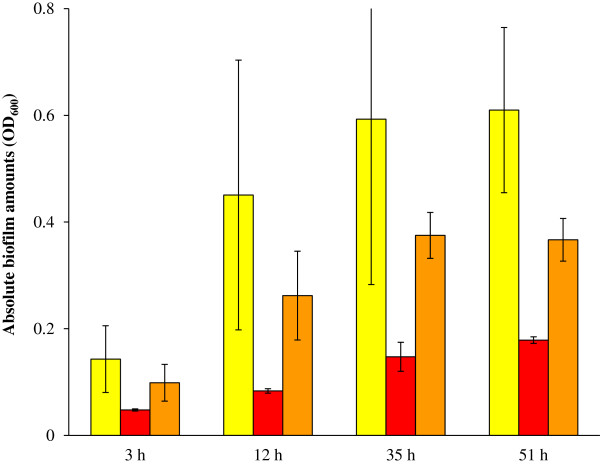
**CV assay to quantify the biofilm amounts of the *ompR *and *rcsB *mutants in comparison to the parent strain.** The biofilm biomass was determined for BP1470 (AJW678 pPS71), BP1531 (*ompR*::*Tn*10 pPS71) and BP1532 (*rcsB*::*Tn*5 pKK12). This was done at four different time points, which are indicated on the x-axis. The yellow bars are the biofilm biomass of the parent strain, the red bars are for the *ompR* mutant, and the orange bars are for the *rcsB* mutant. Averages and standard deviations were calculated across three replicate experiments.

The 3D reconstructions of the biofilms showed that the biofilm from the *ompR* and *rcsB* mutants was much thinner than that of the parent strain. The mutant biofilms were no more than 4 μm, as opposed to >8 μm for biofilm from the parent strain (notice x-axis of Figure 4C versus that of Figure 3C). This observation indicates that the elevation of *flhD* expression levels in the two mutants does indeed have the predicted outcome of reducing biofilm amounts. However, we were unable to quantify thickness of the parental biofilm with the fluorescence microscopy beyond 8 μm due to optical limitations of the objective used for these experiments. To quantify biofilm biomass, the crystal violet (CV) assay was performed with parent bacteria, and *ompR* and *rcsB* mutants (Figure 5). Both mutants produced a considerably smaller amount of biofilm than the parent. This difference was more pronounced for the *ompR* mutant (red bars) than for the rcsB mutant (orange bars).

## Discussion

In the Introduction, we postulated that a biofilm prevention target would be characterized by its expression early in biofilm development. This was the case for *flhD* whose expression peaked at 12 h. A biofilm treatment target was postulated to be characterized by expression late in biofilm development and at the outermost edge of the biofilm. This, too, was true for FlhD/FlhC. Expression of *flhD* increased again towards 51 h, the highest expression of *flhD* was in the outer layer of the biofilm. Based upon these results, we come to the conclusion that the flagella master regulator complex FlhD/FlhC may be our first target for both, biofilm prevention and treatment techniques. This would fulfill our first two goals: i) provide proof of concept that our approach can identify targets for biofilm prevention and treatment techniques and ii) establish FlhD/FlhC as the first such target. In fulfillment of the final goal of this study, we identified two mechanisms to increase *flhD* expression and reduce biofilm amounts. Mutations in the two-component response regulator genes *ompR* and *rcsB* increased *flhD* expression to the point where temporal and spatial differences in expression were abolished. These expression increases where paralleled by decreases in biofilm amounts, relative to the parent strain.

### The expression profiles of *flhD, ompR*, and *rcsB* can be related to Biofilm phases

Originally described in *Pseudomonas aeruginosa,* it is now widely accepted that biofilm development in many bacteria involves reversible attachment, irreversible attachment, maturation, and dispersion [31]. These phases are characterized by cell surface organelles such as flagella, type I fimbriae and curli, as well as numerous exopolysaccharides. The following three paragraphs relate the temporal expression profiles of *flhD* (positive regulator of flagella), *ompR* (negative regulator of flagella and positive regulator of curli), and *rcsB* (negative regulator of flagella and positive regulator of type I fimbriae and colanic acid capsule) to current literature on biofilm developmental phases. According to our previous review [23], the hypothesis for the temporal expression profiles was that *flhD* expression may peak during reversible attachment, *ompR* expression during irreversible attachment, and *rcsB* expression may increase towards maturation.

A recent review article summarized the regulation of motility during biofilm formation [32]. The authors believe that flagella are important in the motility-to-biofilm transition in a way that inhibition of motility encourages biofilm formation by means of several functional (e.g. YcgR) and regulatory (e.g. RcsB) mechanisms [22,33,34]. Our temporal expression profile of *flhD* is partially in agreement with this postulate. We saw a peak in expression at 12 hours (Figure 2), which may resemble reversible attachment, and a time period of low *flhD* expression around 34 h, possibly resembling irreversible attachment. However, expression of *flhD* increased again towards 51 h (Figure 2). This late increase is not necessarily in agreement with current biofilm models. However, Guttenplan and Kearns [32] leave room for flagella regulators that may still be discovered. Also, the role for flagella in dispersal is controversial.

The hypothesis [23] that *ompR* expression may be highest during irreversible attachment was built upon the fact that phospho-OmpR was a negative regulator of *flhD* expression [24] and a positive regulator of curli [28,35]. Our temporal expression profile of *ompR* is in agreement with this hypothesis. The peak for *ompR* was at 34 h, where *flhD* expression was minimal (Figure 2). The production of curli has previously been recognized as a control mechanism for biofilm formation [36], an adherence tool to human uroepithelical cells [37], and part of the motility-to-biofilm transition. CsgD contributes to this transition by activating the expression of curli and inhibiting flagella biosynthesis [38]. The expression peak of the positive curli regulator, OmpR, at 34 h could be our marker for irreversible attachment.

Maturation of a biofilm typically requires the synthesis of an exopolysaccharide capsule that serves as a ‘glue’ to keep the microcolony together and contributes to adherence to the surface. This capsule can consist of many different substances, among them the K-capsule polysaccharide that is a contributor to the intracellular lifestyle of uropathogenic *E. coli*[1] and colanic acid, which has been recognized early as an important factor in forming the three dimensional structures that constitute the biofilm [39]. The phosphorelay system RcsCDB is an activator of colanic acid production [40], while also activating the synthesis of type I fimbriae [25]. These multiple functions of RcsB may explain the slow and steady increase of *rcsB* expression during biofilm formation (Figure 2) that cannot be correlated with a single phase of biofilm development. With the exception of the late increase in *flhD* expression, our temporal expression profiles are in agreement with our hypothesis from the review article [23], as well as current literature.

### Regulation of *flhD* by multiple response regulators offers ample opportunity to control biofilm amounts and cell division

Since the goal of our research was to modulate signal transduction pathways and reduce biofilm amounts, the next step after the identification of FlhD/FlhC as our first target would be the attempt to increase *flhD* expression levels, ultimately causing a reduction in biofilm amounts.

The expression of *flhD* is regulated by many environmental and genetic factors. Environmental factors include temperature [41], osmolarity [24], and the nutritional state of the cell [42]. Genetic factors are similarly diverse and include the Catabolite Repressor Protein CRP and the nucleoid associated protein H-NS [43], the transcriptional regulator LrhA [44], the LysR family protein HdfR [33], and the insertion of IS elements into the *flhD* promoter [45-47]. Post transcriptional regulation involves the carbon storage regulator CsrA [48] and a negative regulator of cell motility, YdiV [49]. At the transcriptional level, regulation of *flhD* expression can be accomplished by several of the response regulators of two-component systems, such as RcsB [50], OmpR [24], and QseC [51].

In this study, knock-out mutations in *rcsB* and *ompR* yielded an impressive increase in *flhD* expression in the *ompR* and *rcsB* mutants (Figures 2 and 4). Additionally, expression of *flhD* was not anymore dependent upon the biofilm phase, after the biofilm had formed (Figure 2) or the location of the individual bacterium within the biofilm (Figure 4). The temporal expression profile of *flhD* in the *ompR* mutant is similar to the one that was observed previously in planktonic bacteria [29]. However, in planktonic bacteria, we never observed more than 2 or 3 fold increases in *flhD* expression in the *ompR* mutant, relative to the parent. Considering the fact that the images for *flhD* in the *ompR* mutant had been obtained at a much reduced excitation intensity (10% versus 90% in the parent strain), the difference in *flhD* expression between the two strains must be much higher in biofilm than in planktonic bacteria.

Intriguingly, the *ompR* and *rcsB* mutants are also our first two mechanisms to reduce biofilm amounts by elevating the expression levels of FlhD/FlhC. This observation provides confidence in our conclusion that impacting the signal transduction cascade, consisting of multiple two-component response regulators and FlhD/FlhC can be used to control biofilm amounts. Since the number of two-component systems in *E. coli* is rather large [28] and response regulators respond to a broad range of environmental signals, the two-component signal transduction mechanism offers ample opportunity at controlling bacterial phenotypes and behaviors by deliberately changing the bacterial environment.

## Conclusions

The bacterial species *E. coli* includes many pathogens, in particular biofilm formation [52,53] and prevention [54] in uropathogenic *E. coli* (UPEC) have been researched intensively over the past few years. The goal of this study was to use an *E. coli* K-12 strain as a model to show that the study of temporal and spatial gene expression can lead to the identification of targets for the development of novel biofilm prevention and treatment options. We propose FlhD/FlhC as the first of such targets and OmpR and RcsB as two mechanisms to control this target. Our intention is to identify more of these targets/target mechanisms, using the temporal/spatial gene expression approach on a selection of biofilm associated genes. With respect to FlhD/FlhC, we believe that a gene that is this highly regulated by so many environmental and genetic factors is ideally suited to be controlled by deliberate changes to the environment, through a signal transduction cascade that may involve additional two-component response regulators beyond OmpR and RcsB, ultimately impacting biofilm amounts. The two-component control mechanism may be particularly important for UPEC strains where two-component signaling plays a large role in motility, quorum sensing, biofilm formation, and virulence [55,56].

## Methods

### Bacterial strains, plasmids, and growth conditions

All the bacterial strains and plasmids that are used for this study are listed in Table 1. Throughout the study, we use the *E. coli* K-12 strain AJW678 as a parental strain because it is a good biofilm former [57] and wild-type for the biogenesis of flagella and type I fimbriae and curli. AJW678 is lacking the IS element [42] in the *flhD* promoter that makes bacteria highly motile. MC1000 is another K-12 strain [58,59]. It contains an IS5 in the *flhD* promoter [47], is highly motile, but produces much reduced biofilm amounts. To assure maximal expression of *flhD*, we use this promoter to construct the *flhD*::*gfp* fusion plasmid pPS71.

**Table 1:** Bacterial strains and plasmids used for this study

**Strains**	**Relevant genotypes**	**Reference**
AJW678	*thi-1 thr-1(am) leuB6 metF159(Am) rpsL136 ΔlaxX74*	[57]
AJW2050	AJW678 *ompR::Tn10*	[42]
AJW2143	AJW678 *rcsB::Tn 5*	[60]
MC1000	F-, *araD*139 Δ(*araAB leu*)7696 Δ(*lacX74*) *galU galK strA prsL thi*	[59]
BP1470	AJW678 pPS71	This study
BP1531	AJW2050 pPS71	This study
BP1532	AJW2143 pKK12	This study
BP1432	AJW678 *ompR::gfp*	This study
BP1462	AJW678 pEC2	This study
BP1437	AJW678 *aceK*::*gfp*	This study
**Plasmids**		
pPS71	pUA66 *flhD::gfp*	This study
pKK12	pPS71 Cm^R^	This study
pOmpR::*gfp*	pUA66 *ompR::gfp*	[62]
pEC2	pAcGFP *rcsB::gfp*	This study
pAceK::*gfp*	pUA66 *aceK*::*gfp*	[62]

The *Tn*10 and *Tn*5 transposons confer resistance towards tetracycline and kanamycin, respectively. Δ constitutes a deletion of the respective gene. Cm^R^ indicates chloramphenicol resistance. *gfp* encodes green fluorescence protein.

AJW2050 is an *ompR* mutant strain due to the insertion of a *Tn*10 transposon [42], AJW2143 is an *rcsB* mutant strain due to *Tn*5 insertion [60]. AJW678, AJW2050, and AJW2143 were kindly provided by Dr. Alan J. Wolfe (Loyola University Chicago, Maywood IL) and used in several of our previous studies [42,61]. Plasmids pPS71 (*flhD::gfp*), pKK12 (pPS71 Cm^R^) and pEC2 (*rcsB::gfp*) were constructed for this study. The *ompR::gfp* plasmid was obtained from the Open Biosystems promoter collection [62] (Thermo Scientific, Huntsville, AL). As a housekeeping gene, we used *aceK* which encodes isocitrate dehydrogenase. This gene was selected because genes encoding enzymes of the tricarboxylic acid cycle have previously been shown to be uniformly expressed in biofilms of *Geobacter sulfurreducens*[11]. In addition, expression from the *aceK*::*gfp* fusion was reasonably steady in a temporal expression experiment with planktonic bacteria (Wilson T., and B.M. Prüß, unpublished data). The *aceK*::*gfp* fusion plasmid was also part of the Open Biosystems promoter collection.

### Cloning of *flhD::gfp* (pPS71), pPS71 Cm^R^ (pKK12) and *rcsB::gfp* (pEC2) plasmids

#### pPS71

To construct the *flhD*::*gfp* containing plasmid, the *flhD* promoter region that starts 1,419 bp upstream of the +1 transcriptional start site and ends 502 bp downstream of the +1 was amplified from MC1000, using 5′-TCCTCGAGTGACTGTGCGCAACATCCCATT-3′ as forward primer and 5′-AGGTACCTGCCAGCTTAACCATTTGCGGA-3′ as reverse primer. This promoter fragment contains the IS5 that increases *flhD* expression and is located at −1,294 bp to −94 bp [47], making the fragment 1,921 bp in length. The forward and reverse primers were designed with XhoI and BamHI restriction enzyme recognition sites at the 5′ ends. The *flhD* promoter fragment was then digested with XhoI and BamHI. The vector pUA66 (Open Biosystems, Huntsville, AL), containing *gfpmut2* as a reporter gene and a kanamycin resistance cassette, was also digested with these enzymes. To reduce re-ligation of the plasmid, digested pUA66 vector was treated with Calf Intestinal Alkaline Phosphatase (CIAP, Promega, Madison WI) that removes the 5′ phosphate. The double digested *flhD* promoter region was ligated into the digested and CIAP-treated pUA66 vector. Competent JM109 cells (Promega, Madison WI) were transformed with the resulting plasmid pPS71. The insertion was confirmed by restriction digest and sequencing. Ultimately, pPS71 was transformed into chemically competent AJW678 and AJW2050.

#### pKK12

The antibiotic resistance of pPS71 was changed from Km^R^ to Cm^R^ creating pKK12. This permitted transformation of the *flhD*::*gfp* fusion plasmid into Km^R^ mutants. pPS71 was digested with EagI to remove 280 bp from pPS71. This deleted region started upstream of the *flhD* promoter and extended upstream into the kanamycin resistance gene. This caused inactivation of kanamycin resistance. The digested plasmid was blunt ended with Klenow (Promega, Madison WI), and treated with CIAP. pHP45Ω-Cm was the source of the chloramphenical resistance gene cassette [63] and was digested with EcoRI and blunt ended with Klenow. The CIAP-treated pPS71 and pHP45Ω-Cm DNA fragments were ligated. Competent JM109 were transformed with the resulting plasmid pKK12, transformants were resistant to chloramphenicol, but not to kanamycin. Competent AJW2143 (*rcsB*::*Kn*) were then transformed with pKK12.

#### pEC2

To construct this plasmid, the *rcsB* promoter region that starts 100 bp upstream of its +1 transcriptional start site and ends 50 bp downstream was PCR-amplified from AJW678, using 5′-GAGAGATCTGCAACCTGTATCACACCCGATGAAAG-3′ as forward primer and 5′-GCAAAGCTTCGGATGGTCATCGGCAATAATTACG-3′ as reverse primer. The PCR-amplified region was then cleaned up and ligated into pGEM-T Easy (Promega, Madison WI). Successful ligations were identified by white color of the transformed colonies. Plasmids were digested using the HindIII and BglII restriction sites that had been added to the 5′ends of the primers. The promoterless pAcGFP1-1 encodes the green fluorescent protein AcGFP1, a derivative of AcGFP from *Aequorea coerulescens,* and has a kanamycin resistance gene (Clontech, Mountain View, CA). This plasmid was also double digested with the same enzymes. The digested *rcsB* promoter region was ligated into the digested pAcGFP1-1 vector. Competent JM109 cells were transformed with the resulting plasmid pEC2. The insertion region was confirmed by restriction digest and sequencing. Ultimately, pEC2 was transformed into chemically competent AJW678.

Bacterial strains were stored at −80°C in 10% dimethyl sulfoxide (DMSO). Before use, the bacterial strains were streaked onto LB (1% tryptone, 0.5% yeast extract, 0.5% NaCl) agar plates and incubated overnight at 37°C. From the plates, cultures were inoculated into liquid tryptone broth (TB, 1% tryptone, 0.5% NaCl) and grown overnight at 37°C. For bacterial strains containing pPS71, 25 μg/ml of kanamycin were added to the bacterial growth medium. For pEC2, 50 μg/ml of kanamycin were added. For pKK12, 50 μg/ml of chloramphenicol were added.

### Temporal and spatial expression of *flhD*, *ompR*, and *rcsB*

*E. coli* strains were grown in TB overnight at 37°C. 1 ml of each culture was injected into one channel of a 3 channel flow cell (Stovall, Greensboro NC) with a syringe as described [8]. The flow cell was incubated at room temperature for one hour without any media flow. After that, TB was pumped by an Isma Tec Low Flow High Accuracy Peristaltic Pump (Stovall) into the flow cell at 1 ml/min, equaling 0.33 ml/min per channel. For temporal expression experiments, the flow cell was disconnected after a maximum of 62 h. For spatial expression experiments, the flow cell was disconnected at time points of interest. Each of the investigated bacterial strains was processed at least three times for both temporal and spatial experiments. The flow cell system was kept free of air bubbles by the bubble trap that is part of the Stovall system.

We used a Zeiss Axio Imager M2 upright fluorescence microscope with ApoTome2 (Zeiss Microimaging, Thornwood NY) to detect the fluorescence signals coming from the promoter::*gfp* fusions. The Zeiss Axio Imager M2 microscope is equipped with a 100×/1.40 oil Paln-Apochromat objective, a Colibri2 higher power LED light source, and a high-resolution monochrome camera for optimal illumination and imaging. For the temporal experiment, fluorescence images were taken at appropriate time points. For the spatial experiments, 20 *z*-stacking images were taken at one or two time points, separately for fluorescence and bright field. Due to the objective working distance limit, *z*-sections could be effectively imaged across 8 μm in depth. In cases where biofilms were thicker than 8 μm on some areas of the slides, we selected areas of the biofilm that were consistent with the limitation of the objective.

The intensities of the fluorescence signals from *aceK*::*gfp* and from *flhD*::*gfp* in the *ompR* and *rcsB* mutants turned out to be much higher than those from the remaining strains and fusions. For this reason, we performed microscopy for BP1437 at 5% of the available excitation light and for BP1531 and BP1532 at 10%. For BP1470, BP1432, and BP1462, we used 90% of the available excitation light.

### Quantification of the fluorescence signals and statistical analysis

To quantify the fluorescence signals that were visualized in the images, we used Image-Pro Plus software and determined the percent area of the image that produced a fluorescent signal. Specifically, pixels values from each image were divided by the pixel values that represent the total area of an image. Under the settings that were used for our imaging, this was 42,100 pixels. Resulting values were multiplied by 100 to yield percent. Next, we determined the average and standard deviation across all 9 images (3 images per biological replicate) for BP1531, BP1532, BP1462, and BP1437 and across the 4 images (1 image from each biological replicate) for BP1470 and BP1432 that were obtained at each time point. Finally, the average percent area was plotted against time for the temporal experiment.

Statistical analysis of the temporal data was done with local regression via the Loess procedure [64]. At each time point, a weighted least squares regression polynomial was fitted to a subset of the data to yield a Loess curve. Confidence bands were computed at a 95% confidence interval. This was done independently for the pPS71 containing parent strain and its *ompR* and *rcsB* mutant strains. To compare temporal expression profiles, overlaps of the confidence bands were determined. A lack of overlap between the confidence bands of any two strains is indicative of a statistically significant difference between the strains. The statistical analysis was done with SAS version 9.2.

For spatial gene expression experiments, 3D reconstructions of the biofilms were done from the *z*-stacked images with AxioVision v-4.7.1 software from Zeiss, using both fluorescence and bright field images. Quantification of the fluorescence signals from these images was done as described for the temporal experiment.

### Crystal violet assay to determine biofilm biomass

Biofilm of BP1470, BP1531, and BP1532 were grown in individual wells of a 24 well plate in TB for 3 h, 12 h, 35 h, and 51 h at room temperature. Liquid bacterial growth medium was removed and biofilms were washed twice with phosphate buffered saline (PBS). Biofilms were stained with crystal violet (CV) as described [65-68]. The OD_600_ of the extracted CV was determined from a 1:10 dilution with a Synergy H1 plate reader from BioTek (Winooski, VT). Averages and standard deviations were determined across the three replicate experiments.

## Abbreviations

CV: crystal violet; GFP: green fluorescence protein.

## Competing interests

The authors declare that they have no competing interests.

## Authors’ contributions

PS constructed the *flhD*::*gfp* plasmid pPS71, performed the fluorescence microscopy, and analyzed the data. He also wrote the first draft of the manuscript. ERC constructed the *rcsB*::*gfp* plasmid pEC2, KK changed the kanamycin resistance of pPS71 to chloramphenicol to yield plasmid pKK12. SMH designed the cloning strategies for all plasmids and supervised the undergraduate students. BMP designed the project, helped PS to set up the flow cells and the microscopy, and contributed to the analysis and interpretation of the data. All authors read the manuscript, made suggestions for changes, and approved the final manuscript.

## Authors’ information

PS is a Ph.D. student in the Molecular Pathogenesis program and the main student working on this NIH funded project. ERC and KK were undergraduate researchers in the Prüß lab. SMH is the research associate in the lab. BMP is the principal investigator of the lab.
